# Reduction of SARS-CoV-2 intra-household child-to-parent transmission associated with ventilation: results from a case–control study

**DOI:** 10.1186/s12889-023-16144-2

**Published:** 2023-06-26

**Authors:** Simon Galmiche, Tiffany Charmet, Yoann Madec, Arthur Rakover, Laura Schaeffer, Olivia Chény, Faïza Omar, Sophie Martin, Alexandra Mailles, Fabrice Carrat, Arnaud Fontanet

**Affiliations:** 1 Emerging Diseases Epidemiology Unit, Institut Pasteur, Université Paris Cité, 25 rue du Docteur Roux, Paris, 75015 France; 2 Sorbonne Université, Ecole Doctorale Pierre Louis de Santé Publique, Paris, France; 3 Institut Pasteur, Université Paris Cité, Centre for Translational Research, Paris, France; 4 Institut Ipsos, Paris, France; 5 Caisse Nationale de L’Assurance Maladie, Paris, France; 6 Santé Publique France, Saint-Maurice, France; 7 Sorbonne Université, Inserm, IPLESP, Hôpital Saint-Antoine, AP-HP, Paris, France; 8 Conservatoire National Des Arts Et Métiers, Unité PACRI, Paris, France

**Keywords:** SARS-CoV-2, Home environment, Family, Patient isolation, Ventilation

## Abstract

**Purpose:**

Our objective was to describe circumstances of SARS-CoV-2 household transmission and to identify factors associated with a lower risk of transmission in a nationwide case–control study in France.

**Methods:**

In a descriptive analysis, we analysed cases reporting transmission from someone in the household (source case). Index cases could invite a non-infected household member to participate as a related control. In such situations, we compared the exposures of the index case and related control to the source case by conditional logistic regression matched for household, restricted to households in which the source case was a child, and the index case and related control were the infected child’s parents.

**Results:**

From October 27, 2020 to May 16, 2022, we included 104 373 cases for the descriptive analysis with a documented infection from another household member. The source case was mostly the index case’s child (46.9%) or partner (45.7%). In total, 1026 index cases invited a related control to participate in the study. In the case–control analysis, we included 611 parental pairs of cases and controls exposed to the same infected child. COVID-19 vaccination with 3 + doses versus no vaccination (OR 0.1, 95%CI: 0.04–0.4), isolation from the source case (OR 0.6, 95%CI: 0.4–0.97) and the ventilation of indoor areas (OR 0.6, 95%CI: 0.4–0.9) were associated with lower risk of infection.

**Conclusion:**

Household transmission was common during the SARS-CoV-2 pandemic in France. Mitigation strategies, including isolation and ventilation, decreased the risk of secondary transmission within the household.

**Trial registration:**

ClinicalTrials.gov registration number: NCT04607941.

**Supplementary Information:**

The online version contains supplementary material available at 10.1186/s12889-023-16144-2.

## Introduction

The household transmission of SARS-CoV-2 has been a crucial issue since the emergence of the disease, given the difficulty of isolating cases from other household members. Studies on household transmission have reported increasing secondary attack rates (SARs) with the emergence of more transmissible variants of concern (VOCs). An early meta-analysis found the SAR to be 16.6% before the emergence of VOCs [1]. Updated estimates for specific VOCs have revealed higher SARs for the B.1.1.7 (alpha), B.1.351 (beta) and B.1.617.2 (delta) VOCs (ranging from 21.0% to 36.4%, depending on the estimate) [2, 3], although temporal differences in prior immunity levels and home isolation recommendations make comparisons difficult. Higher SARs (up to 53%) have been reported for the B.1.1.529 (omicron) variant [2–6]. Comparisons with other settings, such as social events with family and friends have revealed that households are particularly prone to internal transmission events, due to the continuous nature of the exposure of household members in a closed environment [7, 8].

Nevertheless, much less evidence is available concerning the relative contribution of household transmission to the overall SARS-CoV-2 circulation and the circumstances of transmission within households (e.g., relationship between source case and secondary case, isolation measures). Furthermore, there has been little documentation of adherence to isolation measures within households, and few assessments have been made of the efficacy of specific isolation measures, such as mask-wearing, surface disinfection, and the ventilation of indoor areas [4, 9–11].

We aimed to describe circumstances of SARS-CoV-2 household transmission and to identify factors associated with a lower risk of transmission in a nationwide case–control study in France.

## Methods

The methodology of this present case–control study (ComCor project) has been described elsewhere [12–14]. The ComCor study is a nationwide case–control study that began on October 27, 2020. Individuals diagnosed with SARS-CoV-2 infection and, identified through the *Caisse Nationale d’Assurance Maladie* (CNAM, a nationwide public health insurance organization) database, which is notified of all such diagnoses in France, are invited to participate by e-mail. Non-infected controls are selected from a panel representative of the French population by Ipsos, a company specializing in market research and public opinion polls. These controls were not included in the study described here.

### Descriptive analysis

For the descriptive analysis of the present work focusing on household transmission, we selected cases (hereafter referred to as index cases) who reported being infected by another household member, hereafter designated the “source” case, with compatible sequences in symptoms and testing between the participant and that household member.

### Case–control analysis

**Fig. 1:**
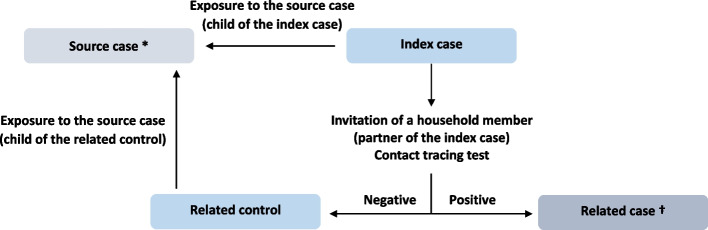
Study design of the inclusion of index cases and related controls for the case–control analysis on household contamination Legend: * The source cases in the household did not participate in the study; †related cases were not included in this analysis; online nationwide study conducted in France between October 2020 and May 2022

After completing the questionnaire, the index cases were offered the possibility of inviting another household member without ongoing SARS-CoV-2 infection (preferably their partner) to participate in the study. They were asked to indicate the e-mail address of the person they wished to invite, and this household member received an e-mail invitation and was asked to provide the result of the test performed during contact tracing to ensure that they remained free of SARS-CoV-2 infection (see Fig. 1). For the case–control analysis, we included invited household members who tested negative during contact tracing, hereafter referred to as “related controls”. We considered adherence to isolation measures and the quality of their implementation to be highly dependent on the relationship between the household members involved. As a means of avoiding highly diverse situations that could not be adequately captured by our questionnaire, and of limiting the risk of unmeasured confounding due to differences in interactions between the source case, the index case and the related control, we restricted the study population to households in which the source case was a child, and the index case and related control were the child’s parents (see Fig. 1).

All participants completed a questionnaire covering sociodemographic characteristics, relationships between household members, exposure information, circumstances of infection, isolation measures, testing and vaccination details. We focused on isolation between the source case and the participant (index case or related control). The question on overall isolation was phrased as follows (English translation of the original French phrasing): “Did you implement isolation measures between yourself and that person [the source of your contamination] (e.g., mask-wearing, door handle disinfection, sleeping separately, etc.)?”. Further questions on specific mitigation measures were phrased as follows: “What isolation measures were implemented? Separate meals, separate toilet and bathroom, mask-wearing, surface and door handle cleaning with a disinfecting product, ventilation of areas for 10–15 min at least twice per day” with a “yes” or “no” answer for each measure. We excluded index cases who were tested or had a symptom onset more than 21 days after testing or symptom onset in the source case. We excluded pairs of index cases and related controls who reported source cases of different ages or sexes. Questionnaire conduction online did not allow to skip any questions, thus we had no missing data.

### Statistical analysis

We used medians and interquartile ranges to describe the population of index cases with a documented case source within the household. We performed univariable and multivariable conditional logistic regression analyses on household-matched cases and controls, with variable selection based on p-values and changes in parameter estimates [15]. We included the following exposures of interest: age, sex, vaccination status (number of doses received: 1, 2, or 3 +), history of past SARS-CoV-2 infection, and underlying conditions (chronic respiratory disease, diabetes mellitus, hypertension, coronary artery disease). We studied the impact of isolating the participant from the source case, by including either overall isolation or individual mitigation measures. We investigated how the time at which transmission occurred might have affected the findings by studying the interaction of the ventilation of indoor spaces with either seasonality or predominance of the omicron strain in two additional multivariable models. We defined the season according to the month in which symptom onset of the source case occurred (or the month of the diagnostic test if the timing of symptom onset was not indicated), with October to March defining the colder months and April to September the warmer months. We defined the time of predominance of the omicron strain from December 20, 2021 until the end of the study [16].

We tested the robustness of our findings in a series of sensitivity analyses. In the first, we fitted a model without any variable selection, i.e., retaining all individual mitigation measures, as well as age, sex, vaccination status, history of infection and underlying conditions [17]. Furthermore, we assessed the risk that unmeasured confounding could explain the association between the main exposures of interest (isolation overall and the mitigation measures) and the risk of infection using the e-value, both for the point estimates and the upper limit of the 95% confidence intervals. The e-value is defined as the minimum strength of association, on a risk ratio scale, that an unmeasured confounder would need to have with the exposure and the risk of infection to fully explain the association [18]. Lastly, to investigate how the selection of households (parental pairs exposed to one infected child) impacted our findings, we performed additional analyses including all eligible households regardless of the relationship between the source case, the index case, and the related control. As for the main analysis, we fitted two models using a variable selection procedure based on p-value and changes in estimates (one including isolation overall and one including individual mitigation measures), and one model without any variable selection, adding a variable defining the relationship between the source case and the participant, categorised as child, parent, sibling, partner, or other.

All analyses were performed with Stata/SE 16.0 software (College Station, Texas, USA).

### Ethical considerations

This study received ethics approval from the *Comité de Protection des Personnes Sud Ouest et Outre Mer 1* on September 21, 2020. The *Commission Nationale de l’Informatique et des Libertés* (CNIL, the French national data protection agency) authorized data processing for this study on October 21, 2020. Informed consent was obtained from all participants. The study is registered with ClinicalTrials.gov under the identifier NCT04607941 (registration date 29/10/2020).

## Results

### Descriptive analysis

**Fig. 2:**
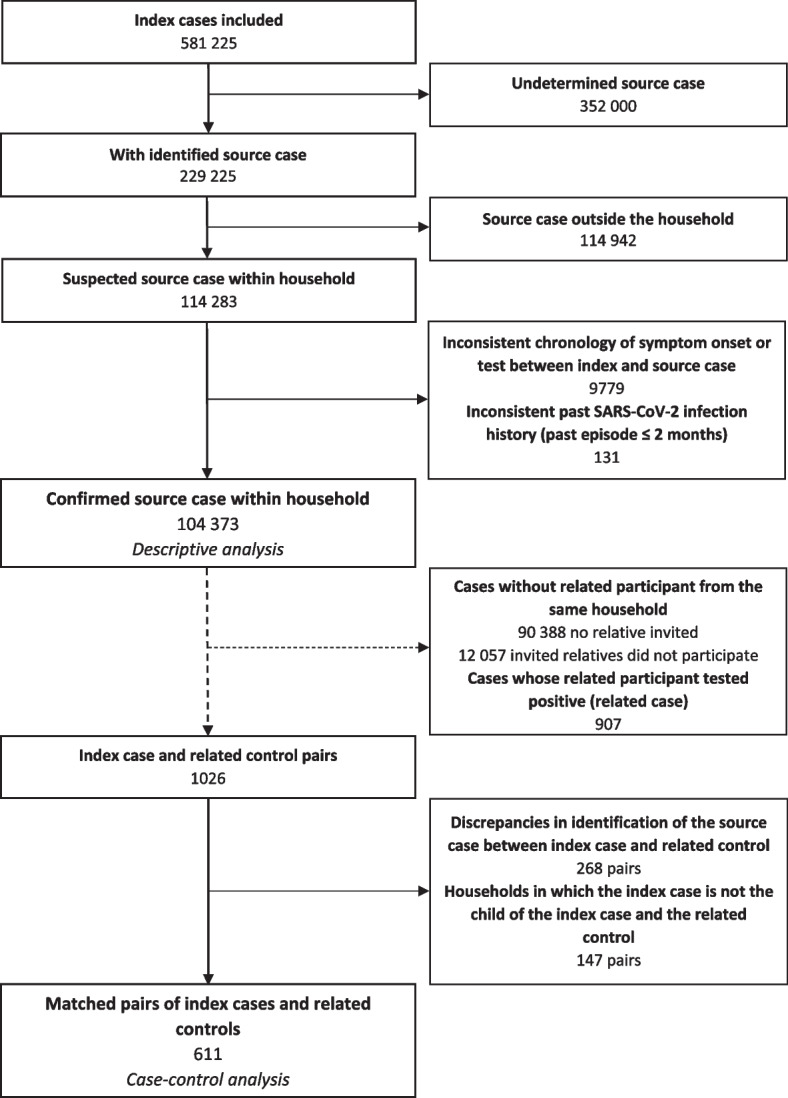
Flow chart for the identification of cases of SARS-CoV-2 infection with a confirmed source case within the household for the descriptive analysis and the selection of pairs of index cases and related controls for the case–control analysis Online nationwide study conducted in France between October 2020 and May 2022

Between October 27, 2020 and May 16, 2022, 581 225 index cases were included in the study, including 229 225 (39.4%) with an identified source of contamination. We included 104 373 index cases with documented intrahousehold contamination for the descriptive analysis. These represent 18.0% of all cases, and 45.5% of those with an identified source of infection (Fig. 2). The main characteristics of the index cases with documented intrahousehold contamination and of the reported source cases are summarized in Table 1. The study population was predominantly female (73.5%), often with a higher education diploma (70.1% with post-secondary education qualifications). The source case was frequently symptomatic (87.5%) and was predominantly the participant’s child (46.9%) or partner (45.7%). Most source cases (54.1%) had isolated themselves from the index case, often after test results (33.8%) or after symptom onset (17.9%). Isolation between the source case and the index case was less frequently implemented when the source case was the index case’s child (50.0%) or partner (55.9%) than when the source case was a sibling (72.4%), mother or father (71.8%) or other member of the household (64.3%) (Pearson’s chi-squared test: *p* < 0.001). Adherence to isolation from the source case decreased over the study period: 57.4% of index cases reported isolation between themselves and the source case in the last quarter of 2020, 63.0% reported such isolation in the first quarter of 2021, decreasing to 47.8% in the first quarter of 2022 (odds test for trend: *p* < 0.001).

**Table 1:** Study population of the descriptive analysis of index cases of SARS-CoV-2 infection with a confirmed source case within their households

**TOTAL**	**104,373**
**Age – median [IQR] (years)**	42 [36–50]
**Female**	76,664 (73.5%)
**Education level**	
No diploma	1687 (1.6%)
Pre-high school diploma	11,778 (11.3%)
High-school diploma	17,513 (16.8%)
Bachelor’s degree	39,281 (37.4%)
Master’s degree or higher	34,114 (32.7%)
**Underlying conditions**	
Chronic respiratory disease	8223 (7.9%)
Hypertension	7541 (7.2%)
Diabetes mellitus	2315 (2.2%)
Coronary artery disease	578 (0.6%)
**COVID-19 vaccination status**	
Unvaccinated	40,529 (38.8%)
1 dose	2294 (2.2%)
2 doses	26,597 (25.5%)
3 doses	34,752 (33.3%)
4 doses	201 (0.2%)
**Symptomatic COVID-19**	90,371 (86.6%)
**Variant**	
Original strain	3219 (3.1%)
Alpha	10,625 (10.2%)
Beta/Gamma	916 (0.9%)
Delta	10,098 (9.7%)
Omicron	12,511 (12.0%)
Other	144 (0.1%)
Undetermined	66,860 (64.1%)
**Number of household members (median [IQR])**	4 [3, 4]
**SOURCE CASE**	
**Age – median [IQR] (years)**	26 [11–45]
**Female**	41,245 (39.5%)
**Symptomatic**	91,302 (87.5%)
**Isolation overall from the index case**	56,415 (54.1%)
**Isolation timing**	
From symptom onset	18,688 (17.9%)
From test results	35,278 (33.8%)
Other timing	2449 (2.4%)
**Mitigation measures**	
Ventilation (10–15 min at least twice/day)	52,150 (50.0%)
Mask-wearing	45,310 (43.4%)
Surface disinfection	41,734 (40.0%)
Separate bathrooms	21,970 (21.1%)
Separate meals	40,706 (39.0%)
**Relationship of the source case to the index case**	
Child	48,971 (46.9%)
Partner	47,726 (45.7%)
Mother/father	3013 (2.9%)
Sibling	1462 (1.4%)
Other	3201 (3.1%)

Legend: Online nationwide study conducted in France between October 2020 and May 2022. Doses of COVID-19 vaccine were counted if they occurred at least 14 days before symptom onset (or testing if asymptomatic) for the first dose, or at least 7 days before symptom onset or testing (if asymptomatic) for the second, third or fourth dose

### Case–control analysis

A small proportion (13,990/104373, 13.4%) of the index cases invited a household member to participate in the study and 1933 (13.8%) of the invited relatives participated in the study. After excluding related participants who subsequently tested positive (related cases, 903), we identified a related control for 1026 index cases. After the elimination of pairs with discrepancies in source case identification and the selection of pairs in which the index case was a parent of the source case and the partner of the related control, we retained 611 matched pairs of index cases and related controls for the case–control analysis (Fig. 2), of whom 597 (97.7%) index cases reported that the source case tested positive for SARS-CoV-2. SARS-CoV-2 infection was confirmed (for cases) or excluded (for controls) using an RT-PCR in respectively 332 (54.3%) and 325 (53.2%) participants (others had a rapid antigenic test – information is missing for 48 cases and 48 controls included before January 2021 when rapid antigenic tests were largely unavailable). The median age of the source case was 11 years (IQR 8–16). For the index cases whose source case was their own child, we found few differences between the 611 index cases with a related control and the overall population of 48,971 index cases included in the main study (online resource, Table S1): the population with a related control was less predominantly female (64.5% vs. 77.1% overall), had a higher education level (80.6% with post-secondary education vs. 70.2%), and reported higher levels of adherence to isolation from the source case, both overall (56.8% vs. 50.0%) and for specific mitigation measures (e.g., 52.5% for the ventilation of indoor areas vs. 46.4%). Differences were otherwise minimal.

The characteristics of index cases and related controls, and conditional logistic regression in univariable and multivariable analyses are described in Table 2. The median time between the participation of the index case and the participation of the related control was 2 days (interquartile range (IQR): 1–4 days). Related controls tested negative for SARS-CoV-2 in median 2 days (IQR 0–4 days) and participated in median 10 days (IQR 7–14 days) after symptom onset in the index case (or test if asymptomatic). There was a strong female preponderance among index cases (64.5% of index cases vs. 35.8% of related controls were women).

**Table 2:** Description of pairs of index cases and related controls and factors associated with the risk of SARS-CoV-2 infection within the household in a household-matched conditional logistic regression analysis

	**Index cases**	**Related controls**	**Univariable analysis**		**Multivariable analysis**					
					**Model with variable selection including isolation overall only**		**Model with variable selection including specific mitigation measures**		**Model without variable selection and including specific mitigation measures**	
	***n* = 611**	***n* = 611**	**Odds ratio**	**95% CI**	**Odds ratio**	**95% CI**	**Odds ratio**	**95% CI**	**Odds ratio**	**95% CI**
**Age, median [interquartile range] (years)**	44 [40–49]	45 [40–50]								
18–39 years	143 (23.4%)	146 (23.9%)	1	-	1	-	1	-	1	-
40–59 years	448 (73.3%)	438 (71.7%)	1.1	0.7–1.8	1.5	0.9–2.5	1.5	0.9–2.5	1.4	0.8–2.5
≥ 60 years	20 (3.3%)	27 (4.4%)	0.5	0.2–1.5	1.2	0.4–3.7	1.2	0.4–3.8	1.2	0.4–3.7
**Female**	394 (64.5%)	219 (35.8%)	1.8	1.5–2.1	1.9	1.5–2.2	1.9	1.5–2.2	1.9	1.6–2.3
**Vaccination status**										
Unvaccinated	181 (29.6%)	171 (28.0%)	1	-	1	-	1	-	1	-
1 dose	15 (2.5%)	20 (3.3%)	0.4	0.1–1.1	0.6	0.2–1.7	0.6	0.2–1.7	0.6	0.2–1.8
2 doses	195 (31.9%)	161 (26.4%)	0.5	0.2–1.2	0.5	0.2–1.4	0.5	0.2–1.4	0.5	0.2–1.3
3–4 doses^**a**^	220 (36.0%)	259 (42.4%)	0.2	0.1–0.4	0.1	0.04–0.4	0.1	0.04–0.3	0.1	0.04–0.3
**History of prior SARS-CoV-2 infection**	26 (4.3%)	52 (8.5%)	0.2	0.1–0.5	0.1	0.1–0.3	0.1	0.1–0.3	0.1	0.1–0.3
**Underlying conditions**										
Chronic respiratory disease	33 (5.4%)	40 (6.6%)	0.8	0.5–1.3	-	-	-	-	0.8	0.4–1.3
Hypertension	50 (8.2%)	44 (7.2%)	1.2	0.7–1.8	-	-	-	-	1.7	1.0–2.8
Diabetes mellitus	5 (0.8%)	14 (2.3%)	0.3	0.1–0.9	-	-	-	-	0.4	0.1–1.2
Coronary artery disease	4 (0.7%)	4 (0.7%)	1	0.3–4.0	-	-	-	-	1.8	0.4–7.7
**Isolation from the source case**										
Isolation overall	347 (56.8%)	378 (61.9%)	0.6	0.4–0.8	0.6	0.4–0.97	-	-	-	-
**Specific mitigation measures**										
Ventilation of indoor areas	321 (52.5%)	350 (57.3%)	0.6	0.4–0.9	-	-	0.6	0.4–0.9	0.5	0.3–0.9
Mask-wearing	305 (49.9%)	318 (52.1%)	0.8	0.5–1.1	-	-	-	-	1.0	0.6–1.8
Surface disinfection	258 (42.2%)	255 (41.7%)	1.1	0.7–1.5	-	-	-	-	1.6	1.0–2.6
Separate bathrooms	165 (27.0%)	176 (28.8%)	0.8	0.5–1.2	-	-	-	-	0.9	0.6–1.5
Separate meals	274 (44.8%)	279 (45.7%)	0.9	0.5–1.4	-	-	-	-	1.2	0.6–2.2

Legend: a: only 3 participants (2 controls and 1 case) had received four doses of COVID-19 vaccines. We therefore grouped these participants with those who had received 3 doses; CI: confidence interval

After matching for household and adjustment for age and sex, a history of past SARS-CoV-2 infection (OR 0.1, 95% CI: 0.1–0.3) and COVID-19 vaccination (for 3–4 doses versus no vaccination, OR 0.1, 95% CI: 0.04–0.4) were associated with a lower risk of infection (Table 2). Overall isolation from the source case was also associated with a lower risk of infection (OR 0.6, 95% CI: 0.4–0.97). The e-values for overall isolation were 2.7 for the point estimate and 1.2 for the upper limit of the confidence interval. We investigated isolation in more detail, by introducing individual mitigation measures into a separate model that did not include overall isolation: the ventilation of indoor areas (OR 0.6, 95% CI: 0.4–0.9) was significantly associated with a lower risk of infection, whereas none of the other components of isolation — mask-wearing, surface disinfection, the use of separate bathroom or eating separately — remained in the model following variable selection (Table 2). The e-values for ventilation were 3.4 for the point estimate and 1.5 for the upper limit of the confidence interval. Source case infection occurred in the colder months for 527 households (86.3%) and in the warmer months for 84 households (13.7%). When we considered the interaction of ventilation with seasonality and with the predominance of the omicron strain, we found that ventilation was associated with a lower risk of infection for exposure during the colder months (OR 0.6, 95% CI: 0.4–0.9) but not during the warmer months (OR 1.1, 95% CI 0.4–3.3), and the association was significant before the emergence of omicron (OR 0.5, 95% CI 0.3–0.9) but not afterwards (OR 0.9, 95% CI 0.5–1.6), although neither of the interaction terms were significant (*p* = 0.220 and *p* = 0.179 for seasonality and omicron predominance, respectively).

In a sensitivity analysis fitting a conditional logistic regression model without variable selection, ventilation of indoor areas remained the only isolation measure significantly associated with a lower risk of infection (OR 0.5; 95% CI: 0.3;0.9) (Table 2) (e-values 3.4 for the point estimate and 1.5 for the upper limit of the confidence interval). When we included all households regardless of the relationship between the source case, the index case, and the related control, we found comparable results for the variables of interest, in particular ventilation of indoor areas: OR 0.7 (95% CI 0.5–0.9) in the model with variable selection, and OR 0.6 (96% CI 0.4–1.0) in the model without variable selection (see online resource, Table S2 for further results).

## Discussion

This study highlights the frequency of household transmission of SARS-CoV-2 in France and the reduction of the risk of transmission within the household associated with isolation measures, particularly the strict application of ventilation measures for indoor areas.

The key role of household transmission has already been reported [7, 8, 19]. We found that household transmission represents a significant proportion of SARS-CoV-2 infections: cases infected within the household represented 18.0% of all cases, likely an underestimation as some household transmissions probably remained undetected, and 45.5% of those reporting an identified source. This underscores the importance of preventing household transmission for comprehensive mitigation policies. Partners and children were the most frequent (over 90%) source of contamination, mirroring the situation of household in France, but this finding may also reflect the poorer application of isolation measures when the source case is a partner or a child, resulting in higher levels of transmission [20].

Adherence to the overall isolation of cases within the household decreased considerably over the study period, probably due to increasing vaccine coverage, and possibly due to some degree of pandemic fatigue. When implemented, isolation from the source case was more frequently applied following test results than at symptom onset, in both index cases and related controls. These findings raise concerns about compliance with public health measures regarding case isolation within households. They also indicate potential targets for improving campaigns for preventing household transmission.

Our analysis of specific mitigation measures highlighted the importance of ventilating indoor areas. We found an interaction of the effect of ventilation with seasonality, with a strong association restricted to the colder months and no significant association during the warmer months. Windows are more likely to be opened in the warmer months, regardless of the infection status of household members, lessening the difference between cases and controls. More generally, while it is conceivable that two adults interacted somewhat differently with the sick child, including on the habits of window opening (when visiting the child’s bedroom for instance), we cannot rule out the possibility that some mitigation measures, including ventilation, had at least a partial effect on the related control when applied by the index case or vice versa. If such an effect occurred, it would have led to an underestimation of the association between various mitigation measures and the risk of infection, thus the magnitude of the odds ratios should be interpreted cautiously. Studies comparing transmission between households might help further assess the impact of these household-level measures on secondary transmission. The fact that ventilation nevertheless remained associated with a lower risk of infection, the sensitivity analysis identifying ventilation as the only significant mitigation measure in a model without variable selection, and the results showing that this effect predominated in the colder months increase our confidence in these findings concerning the importance of ventilation.

Published evidence concerning the efficacy of ventilating indoor areas in the household is particularly scarce. One case–control study in Spain found lower odds of secondary transmission in households with a temperature deemed pleasant despite ventilation [11]. This approximation of ventilation habits differs from that in our study, but the findings are nevertheless consistent. Other school-based studies have reported lower risk of SARS-CoV-2 transmission in schools where improved ventilation systems have been implemented [21, 22]. The ventilation of indoor areas is an interesting target for strategies aiming to reduce household transmission, as its implementation is not limited by housing capacity. The results of the CoviPrev studies conducted on an iterative basis by Santé Publique France to monitor the level of adherence to various risk mitigation strategies in the general public, suggest that ventilation is implemented less during the winter months (e.g., 36.2% of respondents reported systematically ventilating their dwelling at least twice daily in December 2021, whereas this proportion reached 43.1% in May 2022) [23]. Levels of adherence to this measure nevertheless remain quite low overall. We hope that our results will contribute to the design of evidence-based strategies for increasing the use of ventilation measures, particularly in the colder months.

We found no decrease in the risk of transmission due to other isolation measures, such as mask-wearing or eating meals separately, although the lack of significance may result from low power due to the limited sample size. Mask-wearing has been shown to be associated with a lower risk of infection in a number of studies [10, 11, 24, 25], including one by the US Centers for Disease Control and Prevention in which attack rates were lower in households in which index cases reported wearing a mask during the infectious period (39.5% vs. 68.9%) [4]. Our findings for mask-wearing may also reflect difficulties complying with this measure in the household setting, in which the continual correct wearing of a mask may be particularly arduous.

Women were markedly overrepresented among the cases, consistent with the composition of the case population of the ComCor study: women accounted for 68.8% of all cases included during the study period, but only 53% of all notified cases in France over the same period [26]. There was therefore a selection bias in our study with a corresponding overrepresentation of men in the group of related controls (these controls being the partners of the index cases in this study). The higher risk of household transmission for women should, therefore, be interpreted with particular caution. Interestingly, the proportion of women was higher in the subpopulation of index cases reporting their child as the source case (77.1%) than in the total population of index cases infected within the household (73.5%), potentially reflecting women having more contact than men with their children in French households [27].

This study has several limitations, including the self-declared nature of the data, leaving little possibility for verification. Identification of the source case was likely mistaken in some of the cases, particularly given high share of asymptomatic infections in children and possible multiple exposure [28, 29]. We implemented several coherence checks on contamination chain analysis and adherence to design for the intrahousehold analysis, and almost all index cases indicated that the source case had a positive SARS-CoV-2 test. The description of isolation and mitigation measures had to be kept at a level of detail appropriate for a self-administered questionnaire, which might have particularly affected the question on overall isolation: isolation as a bundle of mitigation measures might be diversely understood. Thus, findings on this item should be interpreted with caution. Index cases had the option of inviting another household member to participate, but there was no obligation. As a result, such invitations occurred in only a small proportion of cases, resulting in a selection of the households included in the case–control analysis. It is difficult to speculate on how this selection process might have affected study findings concerning isolation or the ventilation of indoor areas, for example. The selection of a more female, more educated study population may however limit the generalizability of our results. Furthermore, some descriptive indicators might be overestimated, such as adherence to the mitigation measures, in this selected population.

Owing to the relatively small sample size, we could not investigate all the potential changes of associations through time (for instance depending on non-pharmaceutical interventions or the circulating strain).

Validity of the variable selection procedure in model selection is debated, but our results remained unchanged in the sensitivity analysis based on a fitted model without any variable selection [17].

We had limited data concerning the other members of the household (potentially also infected by SARS-CoV-2) who might represent additional exposures, and impact the associations observed in unpredictable manner. While the e-values for the point estimates are quite high, they are relatively low for the upper limit of the confidence intervals. Although it is unclear which confounding factor would not be accounted for in the analyses, these e-values illustrate the possibility that unmeasured confounding might explain the associations between isolation or ventilation and the risk of infection, stressing the need to confirm these findings in other studies. Finally, we cannot exclude the possibility that the related control was ultimately infected, as related controls tested negative quite early after symptom onset in the index case, and close to 40% of them used a rapid antigenic test which is less sensitive than RT-PCR. However, related controls participated a median 10 days after symptom onset in the index case and were still likely asymptomatic by then as they confirmed absence of transmission.

Overall, this study provides evidence of the prominent role of household transmission in SARS-CoV-2 circulation in France and of the benefits associated with implementing mitigation measures in the household, including the ventilation of indoor areas in particular. These findings will be useful for the design of public health mitigation strategies targeting households and informing the public about the importance of ventilating indoor areas, alongside other pharmaceutical and non-pharmaceutical measures.

## Supplementary Information

**Additional file 1: Table S1.** Index cases reporting household contamination from their child, both overall and restricted to the subpopulation of index cases with a partner participating in this study as a related control. **Table S2.** Sensitivity analysis including in the study population the households other than parental pairs exposed to the same infected child and exposures associated with the risk of SARS-CoV-2 infection.

## Data Availability

The data that support the findings of this study are available at Institut Pasteur. Restrictions apply to the availability of these data, which were used under authorized agreement for this study from the data protection authority, the *Commission Nationale de l’Informatique et des Libertés* (CNIL). Access to these data would therefore require prior authorization by the CNIL. Contact the corresponding author (Arnaud Fontanet, fontanet@pasteur.fr) for requests.
