# Supplementary material for: Reduction of SARS-CoV-2 intra-household child-to-parent transmission associated with ventilation: results from a case–control study
Source: BMC Public Health. 2023 Jun 26;23:1240. doi: 10.1186/s12889-023-16144-2 (PMC10294317; doi:10.1186/s12889-023-16144-2)
Supplement: Supplementary file 1 — Additional file 1: Table S1. Index cases reporting household contamination from their child, both overall and restricted to the subpopulation of index cases with a partner participating in this study as a related control. Table S2. Sensitivity analysis including in the study population the households other than parental pairs exposed to the same infected child and exposures associated with the risk of SARS-CoV-2 infection. [file 12889_2023_16144_MOESM1_ESM.pdf]

Supplementary information

Reduction of SARS-CoV-2 household transmission associated with ventilation: results from a case-control study

BMC Public Health

Simon Galmiche[a, b], Tiffany Charmet[a], Yoann Madec[a], Arthur Rakover[a], Laura Schaeffer[a], Olivia Chény[c], Faïza Omar[d], Sophie Martin[e], Alexandra Mailles[f], Fabrice Carrat[g], Arnaud Fontanet[a, h]

[a] Emerging Diseases Epidemiology Unit, Institut Pasteur, Université Paris Cité, 25 rue du Docteur Roux, 75015 Paris, France

[b] Sorbonne Université, Ecole Doctorale Pierre Louis de Santé Publique, Paris, France

[c] Institut Pasteur, Université Paris Cité, Centre for Translational Research, Paris, France

[d] Institut Ipsos, Paris, France

[e] Caisse Nationale de l'Assurance Maladie, Paris, France

[f] Santé Publique France, Saint-Maurice, France

[g] Sorbonne Université, Inserm, IPLESP, Hôpital Saint-Antoine, AP-HP, Paris, France

[h] Conservatoire National des Arts et Métiers, Unité PACRI, Paris, France

Corresponding author: Arnaud Fontanet, Emerging Diseases Epidemiology Unit, Institut Pasteur, 25 rue du Docteur Roux, 75015 Paris, France

Phone: +33140613763

E-mail: fontanet@pasteur.fr

Table S1: Index cases reporting household contamination from their child, both overall and restricted to the subpopulation of index cases with a partner participating in this study as a related control

	Index cases with their child as the source case	Index cases with their child as the source case and their partner as a related control
**TOTAL**	**48971**	**611**
**Age, median [interquartile range] (years)**	42 [38-48]	44 [40-49]
**Female**	37769 (77.1%)	394 (64.5%)
**Education level**		
No diploma	889 (1.8%)	8 (1.3%)
Pre-high school diploma	5558 (11.4%)	56 (9.2%)
High-school diploma	8156 (16.7%)	54 (8.8%)
Bachelor’s degree	19679 (40.2%)	208 (34.0%)
Master’s degree or higher	14689 (30.0%)	285 (46.6%)
**Underlying conditions**		
Chronic respiratory disease	3987 (8.1%)	33 (5.4%)
Hypertension	3041 (6.2%)	50 (8.1%)
Diabetes mellitus	936 (1.9%)	5 (0.8%)
Coronary artery disease	195 (0.4%)	4 (0.7%)
**COVID-19 vaccination status**		
Unvaccinated	13705 (28.0%)	181 (29.6%)
1 dose	1124 (2.3%)	15 (2.5%)
2 doses	13992 (28.6%)	195 (31.9%)
3 doses	20059 (41.0%)	219 (35.8%)
4 doses	91 (0.2%)	1 (0.2%)
**Symptomatic COVID-19**	43614 (89.1%)	530 (86.7%)
**Variant**		
Original strain	1163 (2.4%)	18 (3.0%)
Alpha	3725 (7.6%)	65 (10.6%)
Beta/Gamma	279 (0.6%)	3 (0.5%)
Delta	5074 (10.4%)	93 (15.2%)
Omicron	6649 (13.6%)	70 (11.5%)
Other	58 (0.1%)	0
Undetermined	32023 (65.4%)	362 (59.2%)
**Number of household members (median [IQR])**	4 [3-4]	4 [3-4]
**SOURCE CASE**		
**Age, median [interquartile range] (years)**	10 [7-16]	11 [8-16]
**Female**	23078 (47.1%)	274 (44.8%)
**Symptomatic**	39903 (81.5%)	508 (83.1%)
**Isolation from the index case**	24459 (50.0%)	347 (56.8%)
**Isolation timing**		
From symptom onset	7737 (15.8%)	88 (14.4%)
From test results	15586 (31.8%)	242 (39.6%)
Other timing	1136 (2.3%)	17 (2.8%)
**Specific mitigation measures**		
Ventilation (10-15 min at least twice/day)	22696 (46.4%)	321 (52.5%)
Mask-wearing	20076 (41.0%)	305 (49.9%)
Surface disinfection	18508 (37.8%)	258 (42.2%)
Separate bathrooms	9713 (19.8%)	165 (27.0%)
Separate meals	18160 (37.1%)	274 (44.8%)

Legend: Doses of COVID-19 vaccine were counted if they occurred at least 14 days before symptom onset (or testing if asymptomatic) for the first dose or at least 7 days before symptom onset or testing (if asymptomatic) for the second, third or fourth dose.

Table S2: Sensitivity analysis including in the study population the households other than parental pairs exposed to the same infected child and exposures associated with the risk of SARS-CoV-2 infection

	Index cases	Related controls	Univariable analysis		Multivariable analysis					
					Model with variable selection including isolation overall only		Model with variable selection including specific mitigation measures		Model without variable selection and including specific mitigation measures	
	Cases	Controls	OR	95% CI	OR	95% CI	OR	95% CI	OR	95% CI
	n=758	*n*=758								
**Age. median (IQR)**	45 (40-51)	44 (38-49)								
18-39 years	182 (24.0%)	251 (33.1%)	1		1		1		1	
40-59 years	541 (71.4%)	464 (61.2%)	2.5	1.8-3.4	1.4	0.9-2.3	1.4	0.8-2.3	1.4	0.9-2.3
≥ 60 years	35 (4.6%)	43 (5.7%)	1.2	0.6-2.4	1.2	0.5-3.0	1.2	0.5-3.0	1.2	0.5-3.0
**Female**	510 (67.3%)	321 (42.3%)	1.8	1.5-2.1	1.8	1.5-2.2	1.8	1.5-2.2	1.9	1.6-2.2
**Vaccination status**										
Unvaccinated	253 (33.4%)	244 (32.2%)	1		1		1		1	
1 dose	18 (2.4%)	25 (3.3%)	0.4	0.2-1.1	0.6	0.2-1.8	0.6	0.2-1.8	0.6	0.2-1.7
2 doses	226 (29.8%)	185 (24.4%)	0.7	0.3-1.4	0.6	0.2-1.4	0.6	0.2-1.4	0.6	0.2-1.4
3-4 doses	261 (34.4%)	304 (40.1%)	0.2	0.1-0.6	0.1	0.04-0.4	0.1	0.04-0.3	0.1	0.04-0.3
**History of prior SARS-CoV-2 infection**	30 (4.0%)	64 (8.4%)	0.2	0.1-0.5	0.1	0.1-0.3	0.1	0.1-0.3	0.1	0.1-0.3
**Underlying conditions**										
Chronic respiratory disease	41 (5.4%)	46 (6.1%)	0.9	0.6-1.4		-		-	0.8	0.5-1.4
Diabetes mellitus	12 (1.6%)	20 (2.6%)	0.6	0.3-1.2		-		-	0.5	0.2-1.2
Hypertension	63 (8.3%)	54 (7.1%)	1.2	0.8-1.8		-		-	1.5	0.9-2.4
Coronary artery disease	5 (0.7%)	4 (0.5%)	1.3	0.3-4.7		-		-	1.7	0.4-7.4
**Mitigation measures from the source case**										
Overall isolation	455 (60.0%)	504 (66.5%)	0.5	0.3-0.7	0.6	0.4-0.8				
**Mitigation measures**										
Ventilation of indoor areas	421 (55.5%)	459 (60.6%)	0.6	0.4-0.8		-	0.6	0.4-0.9	0.6	0.4-1.0
Mask-wearing	403 (53.2%)	425 (56.1%)	0.7	0.5-1.0		-		-	1.0	0.6-1.7
Surface disinfection	339 (44.7%)	344 (45.4%)	0.9	0.7-1.3		-	1.4	0.9-2.1	1.4	0.9-2.2
Separate meals	367 (48.4%)	390 (51.5%)	0.6	0.4-0.9		-		-	0.9	0.5-1.6
Separate bathroom	212 (28.0%)	242 (31.9%)	0.7	0.5-0.9		-		-	0.9	0.6-1.4
**Source case is participant's…**										
Sibling	11 (1.5%)	26 (3.4%)	1		1		1		1	
Partner	65 (8.6%)	4 (0.5%)	24.1	3.0-194.2	14.9	1.8-124.2	17.4	2.1-147.0	13.5	1.5-121.0

Parent	16 (2.1%)	67 (8.8%)	1.6	0.2-10.8	1.0	0.1-7.2	1.1	0.2-8.1	0.9	0.1-6.9
Child	646 (85.2%)	615 (81.1%)	2.5	1.2-5.3	2.5	1.0-6.2	2.6	1.0-6.6	2.4	1.0-6.3
Other	20 (2.6%)	46 (6.1%)	0.6	0.2-1.9	0.9	0.3-3.1	0.9	0.3-3.3	0.8	0.2-3

Legend: Case-control study conducted online in France between October 2020 and May 2022. Index cases were offered the possibility to invite a non-infected member of their household to participate, preferably their partner. Both the index cases and the related control shared the household with a source case (not included in the study). Conditional logistic regression univariable and multivariable models were matched on households.
